# Where the ‘bad’ and the ‘good’ go: A multi-lab direct replication report of Casasanto (2009, Experiment 1)

**DOI:** 10.3758/s13421-024-01637-1

**Published:** 2024-09-23

**Authors:** Yuki Yamada, Jin Xue, Panpan Li, Susana Ruiz-Fernández, Asil Ali Özdoğru, Şahsenem Sarı, Sergio C. Torres, José A. Hinojosa, Pedro R. Montoro, Bedoor AlShebli, Aidos K. Bolatov, Grant J. McGeechan, Mircea Zloteanu, Irene Razpurker-Apfeld, Adil Samekin, Nurit Tal-Or, Julian Tejada, Raquel Freitag, Omid Khatin-Zadeh, Hassan Banaruee, Nicolas Robin, Guillermo Briseño-Sanchez, Carlos J. Barrera-Causil, Fernando Marmolejo-Ramos

**Affiliations:** 1 Faculty of Arts and Science, Kyushu University, Fukuoka, Japan; 2 School of Foreign Languages, Beijing Institute of Technology, Beijing, China; 3 School of Foreign Studies, University of Science and Technology Beijing, Beijing, China; 4 Zibo Zhangdian No. 8 Middle School, Zibo, Shandong Province China; 5 BTU Brandenburg University of Technology, Cottbus-Senftenberg, Germany; 6 Department of Psychology, Marmara University, Istanbul, Türkiye; 7 Department of Psychology, Üsküdar University, Istanbul, Türkiye; 8 Multimodal Interaction Lab, Leibniz Institut für Wissensmedien, Tübingen, Germany; 9 Instituto Pluridisciplinar, Universidad Complutense de Madrid, Madrid, Spain; 10 Dpto. Psicología Experimental, Procesos Cognitivos y Logopedia, Universidad Complutense de Madrid, Madrid, Spain; 11 Centro de Ciencia Cognitiva - C3, Universidad Nebrija, Madrid, Spain; 12 Departamento de Psicología Básica 1, Facultad de Psicología, Universidad Nacional de Educación a Distancia, Madrid, Spain; 13 Social Science Division, New York University Abu Dhabi, Abu Dhabi, United Arab Emirates; 14 School of Medicine, Astana Medical University, Astana, Kazakhstan; 15 Shenzhen University Medical School, Shenzhen University, Shenzhen, China; 16 Centre for Applied Psychological Sciences, Teesside University, Middlesbrough, UK; 17 Department of Criminology, Politics, and Sociology, Kingston University London, London, UK; 18 Department of Behavioral Sciences, Zefat Academic College, Safed, Israel; 19 M. Narikbayev KAZGUU University, Astana, Kazakhstan; 20 Department of Communication, University of Haifa, Haifa, Israel; 21 Department of Psychology, Federal University of Sergipe, São Christóvão, Brazil; 22 Department of Letters, Federal University of Sergipe, São Christóvão, Brazil; 23 School of Foreign Languages, University of Electronic Science and Technology of China, Chengdu, China; 24 University of Education Weingarten, Weingarten, Germany; 25 Faculty of Sport Sciences, Université des Antilles, Pointe-à-Pitre, France; 26 Department of Statistics, TU Dortmund University, Dortmund, Germany; 27 Facultad de Ciencias Exactas y Aplicadas, Instituto Tecnológico Metropolitano, Medellín, Colombia; 28 University of South Australia Online, Adelaide, Australia

**Keywords:** Embodied cognition, Body-specificity hypothesis, Social cognition, Conceptual mapping, Space–valence association, Handedness, Big team science

## Abstract

Casasanto (*Journal of Experimental Psychology: General*, *138*, 351–367, 2009) conceptualised the body-specificity hypothesis by empirically finding that right-handed people tend to associate a positive valence with the right side and a negative valence with the left side, whilst left-handed people tend to associate a positive valence with the left side and negative valence with the right side. Thus, this was the first paper that showed a body-specific space–valence mapping. These highly influential findings led to a substantial body of research and follow-up studies, which could confirm the original findings on a conceptual level. However, direct replications of the original study are scarce. Against this backdrop and given the replication crisis in psychology, we conducted a direct replication of Casasanto’s original study with 2,222 participants from 12 countries to examine the aforementioned effects in general and also in a cross-cultural comparison. Our results support Casasanto’s findings that right-handed people associate the right side with positivity and the left side with negativity and vice versa for left-handers.

## Introduction

The relationship between affectivity and bodily experiences, along with their spatial dynamics, has attracted significant attention in cognitive and affective sciences. Especially in the fields of social psychology and embodied cognition, numerous studies have been conducted to explore this relationship. For example, several studies revealed that the upper space is associated with positivity and the lower space is associated with negativity (e.g., Casasanto & Dijkstra, 2010; Cervera-Torres et al., 2018; Meier & Robinson, 2004; Sasaki et al., 2015, 2016). A potential explanation for these associations can be found in the conceptual metaphor theory (Lakoff & Johnson, 2008). In this regard, it is assumed that bodily manifestations of emotions, like standing upright while feeling happy or adopting a slouched posture when feeling sad, might contribute to the formation of associations between spatial bodily orientation and valence (cf. Cian, 2017). These associations can also be observed in metaphorical representations of abstract experiences in language (e.g., metaphors such as “feeling high” to describe happiness or “feeling down” to describe sadness; cf. Marmolejo-Ramos et al., 2017).

Arguably, one of the most fundamental and influential explanation of space–valence associations can be seen in the body-specificity hypothesis by Casasanto (2009). The body-specificity hypothesis postulates that space–valence associations are body-specific and depend on the dominant hand. Casasanto argued that people can better interact with the environment with their dominant hand, leading to a more fluent interaction with the associated space side. In turn, this higher fluency has been showed to be associated positively (see also Beilock & Holt, 2007; Oppenheimer, 2008; Zhang et al., 2022). To test this assumption, Casasanto developed a series of experiments whose findings supported his hypothesis. In the following, we describe only one of these experiments in more detail (Experiment 1) since this experiment is the subject of our replication.

In Experiment 1 of Casasanto’s (2009) study, participants were given a short story about Bob, a fictional character. One group of participants were told that Bob loves zebras and hates pandas; the other group of participants were told that Bob loves pandas and hates zebras. Then, the participants were asked to draw the animals in a right or a left box. More specifically, subjects were told to draw the “good” animal in the box that represents good things best and the “bad” animal in the box that represents bad things best. It is also important to note that this was a paper–pencil study in which the fictional character, Bob, was shown from a bird’s-eye perspective, and the two boxes were shown on the right and left side of Bob.

The results showed that right-handed participants are more likely to put the “good” animal in the right box and the “bad” animal in the left box, whilst the left-handed participants are more likely to put the “good” animal in the left box and the “bad” animal in the right box. Accordingly, it was concluded that right-handed participants associate the right side with a positive and the left side with a negative valence. Left-handed participants, on the other hand, associate the left side with a positive and the right side with a negative valence. Thus, the findings of Casasanto’s study support his body-specificity hypothesis by showing a body-specific valence mapping.

After this study, the body-specificity hypothesis found conceptual support from a wide array of follow-up studies, deepening the understanding of the underlying mechanisms of these space–valence associations and generalizing them across different action contexts (e.g., Brouillet et al., 2015; Casasanto & Chrysikou, 2011; Casasanto & Henetz, 2012; Casasanto & Jasmin, 2010; Cervera-Torres et al., 2020; de la Fuente, Casasanto, Román et al., 2015a, de la Fuente, Casasanto, Santiago et al., 2015b, 2017; de la Vega et al., 2012, 2013; Kominsky & Casasanto, 2013; Marmolejo-Ramos et al., 2013, 2017).

However, the aforementioned studies, which conceptually support and clarify the left–right valence mappings, cannot be understood as direct replications of Casasanto’s (2009) original study. Only a few studies used a more comparable one-to-one replication approach to the original study. For example, Song et al. (2019, Experiment 1) used a modified version of “Bob’s story,” in which participants wrote the names of the animals into the boxes instead of drawing them. In addition, the panda was replaced by a giraffe. In a similar vein, Brouillet et al. (2015, Experiment 1a) used small plastic animal figures that participants had to place into the boxes in Bob’s story.

Against the backdrop of the aforementioned high relevance of Casasanto’s (2009) study, which has been cited more than 900 times to date, and the small number of one-to-one replications, we deem it fruitful to directly replicate Experiment 1 of Casasanto’s original study with a larger sample. Replicating one of the most influential studies and, therewith, confirming or rejecting one of the most influential theories at the intersection of embodied cognition and social psychology is also supported from a more general perspective, as not many influential papers in the aforementioned area have been sufficiently replicated (cf. Schmidt, 2009; Wiggins & Christopherson, 2019; Zwaan et al., 2017). Thus, a multilaboratory setting, in which data from a larger number of countries can be handled simultaneously, can increase the generalizability of findings (Henrich et al., 2010; Yarkoni, 2022; Zwaan et al., 2017). Our research objective is examining whether right-handed people associate positive valence with the right side and negative valence with the left side and whether left-handed people associate positive valence with the left side and negative valence with the right side.

## Methods

### Participants

Our sample (*n* = 2,222) consists of 154 left-handers and 2,068 right-handers from 12 countries. Table 1 summarizes the demographics of the participants for each country, including the sample size by country, age, gender and handedness. The participants obtained payment or course credits for the participation in the study.

**Table 1:** Demographics (*n* = 2,222)

Country	n	Age		Gender			Handedness	
		Mean	SD	Female	Male	Non-binary	Left	Right
Brazil	154	25.79	8.37	91	55	8	4	150
China	147	20.97	4.08	60	85	2	2	145
Germany	478	26.27	4.52	364	114	0	23	455
Guadeloupe (France)	151	21.05	2.57	60	91	0	18	133
India	163	30.07	7.48	88	75	0	3	160
Iran	165	34.90	10.83	97	68	0	27	138
Israel	159	25.62	4.52	92	67	0	11	148
Japan	161	18.93	1.33	88	72	1	15	146
Kazakhstan	160	24.75	7.99	127	33	0	8	152
Spain	185	23.95	9.43	142	43	0	16	169
Türkiye	141	37.34	10.71	72	69	0	16	125
United Kingdom	158	25.44	10.81	133	24	1	11	147

### Materials

Materials included four versions of Bob’s story. As in the horizontal task in Casasanto’s (2009) Experiment 1, all versions indicated to participants that by flipping the page they would meet Bob, a fictional character that is planning to visit a zoo. In two versions they were told that Bob thinks that pandas were good and zebras were bad animals, and in two versions that Bob thinks that zebras were good and pandas bad. In one of the respective two versions, the bad animal was mentioned first, and in the other version, the good animal was mentioned first. In all four versions participants see a forehead (Bob) and two squares, one on the right and one on the left in front of Bob (see Fig. 1a in Casasanto, 2009) where they have to place the good and bad animals. These four versions ensured that the assignment of valence to the panda and zebra was counterbalanced across participants. The versions also ensured that the associations between valence and space were not confounded with the associations between space and the temporal order of the presentation of the animals by counterbalancing the order of the presentation of the good and the bad animals across participants.

Besides Bob’s story and demographic questions relating to age and gender, participants were asked to complete the FLANDERS handedness survey (Nicholls et al., 2013) in order to distinguish between right-handers and left-handers.

### Procedure

All participants performed the task in their own native language. Participants were assigned randomly and with equal likelihood to one of the four versions of Bob’s story described in the “Materials” section above. As in Casasanto’s (2009) Experiment 1, the task of the participants was to determine the horizontal spatial locations (left, right) of Bob’s loved and hated animals (pandas, zebras) by taking the character’s perspective. To do so, they were told to allocate the good animal in the box that represents good things best and to allocate the bad animal in the box that represents bad things best by writing the first letter of the respective animal (e.g., “p” for panda and “z” for zebra in the English version of the study).

Due to the COVID-19 pandemic and the respective anti-coronavirus measures, half of the participating countries (Türkiye, Spain, Japan, Iran, Guadaloupe, and China) were able to collect the data via paper and pencil, as originally planned. The UK had to implement an online version of the experiment in order to finish data collection (12.42% of the data were collected via paper–pencil, the rest via online). The remaining countries (Brazil, Germany, India, Israel, Kazakhstan) collected the data solely via online. SosciSurvey was used for online data collection.

In the online version of the experiment, participants were told to choose the box on the screen that represents good things for the good animal best and the box that represents bad things for the bad animal best by typing the first letter of the respective animal (e.g., “p” for panda and “z” for zebra in the English version of the study). As both of the letters were assigned to good and bad animals, depending on the version of “Bob’s story” (i.e., panda can be a good or a bad animal and, in turn, zebra can be a good or a bad animal), effects of possible valence–space associations with keyboard letters cannot be expected.

After completing the task, participants were asked to report their handedness, age, and gender.

## Results

**Fig. 1:**
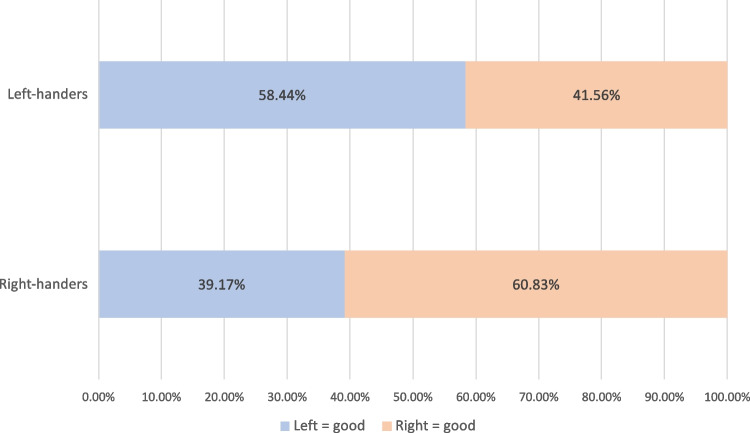
Proportion of left- and right-handers who positioned the good animal in the left box and in the right box (*n* = 2,222)

We performed the same analysis as Casasanto (2009) to allow a comparison of our results with the results of the original study.^1^ As in Casasanto, a majority (58%) of left-handers positioned the good animal in the box on the left of the cartoon character (sign test on 90 left side vs. 64 right side, *p* value = .044), whereas a majority (61%) of right-handers positioned the good animal in the box on the right (sign test on 810 left side vs. 1,258 right side, *p* value = .000; see Fig. 1).

By Fisher’s exact test, there was a significant correlation between the handedness of the participant and the left–right placement of the good and bad animals (*p* value = .004).

The strength of this correlation was evaluated with a binary logistic regression. The odds ratio (*OR*) for the regression of left–right preference on handedness was estimated at 2.18, 95% CI [1.57, 3.06], indicating that right-handers were roughly two times more likely than left-handers to place the good animal on the right and the bad animal on the left.

A second binary logistic regression, with country as a random effect, was performed in order to determine whether the effect of handedness remains significant across countries. The *OR* for the regression of left–right preference on handedness was estimated at 2.10, 95% CI [1.50, 2.95], indicating that right-handers were still roughly two times more likely than left-handers to place the good animal on the right and the bad animal on the left. The descriptive statistics by country are represented in Table 2; inferential statistics were not applied on a per-country analysis, as the number of left-handers in each country is too low for a valid inferential statistical analysis. Please note that the country-focused analysis was not part of Casasanto’s (2009) original study.

**Table 2:** Descriptive statistics by country (*n* = 2,222)

Country	Handedness	n	Freq. of responses		Proportion of responses	
			Good-left	Good-right	Good-left	Good-right
Brazil	Left	4	3	1	75.00%	25.00%
	Right	150	60	90	40.00%	60.00%
China	Left	2	1	1	50.00%	50.00%
	Right	145	62	83	42.76%	57.24%
Germany	Left	23	16	7	69.57%	30.43%
	Right	455	181	274	39.78%	60.22%
Guadeloupe (France)	Left	18	7	11	38.89%	61.11%
	Right	133	45	88	33.83%	66.17%
India	Left	3	1	2	33.33%	66.67%
	Right	160	37	123	23.13%	76.88%
Iran	Left	27	12	15	44.44%	55.56%
	Right	138	77	61	55.80%	44.20%
Israel	Left	11	3	8	27.27%	72.73%
	Right	148	52	96	35.14%	64.86%
Japan	Left	15	8	7	53.33%	46.67%
	Right	146	59	87	40.41%	59.59%
Kazakhstan	Left	8	5	3	62.50%	37.50%
	Right	152	73	79	48.03%	51.97%
Spain	Left	16	13	3	81.25%	18.75%
	Right	169	59	110	34.91%	65.09%
Türkiye	Left	16	14	2	87.50%	12.50%
	Right	125	55	70	44.00%	56.00%
United Kingdom	Left	11	7	4	63.64%	36.36%
	Right	147	50	97	34.01%	65.99%
Total	Left	154	90	64	58.44%	41.56%
	Right	2068	810	1258	39.17%	60.83%

## Discussion

The present study aimed to directly replicate Experiment 1 of Casasanto’s (2009) original study with a larger sample. In order to increase the generalizability of the findings (Henrich et al., 2010; Yarkoni, 2022; Zwaan et al., 2017), we choose a multilaboratory setting. Thus, our research objective was to examine whether right-handed people tend to associate positive valence with the right side and negative valence with the left side and whether left-handed people tend to associate positive valence with the left side and negative valence with the right side.

Our results reveal that roughly 60% of our participant associate positive valence with their dominant side (i.e., roughly 60% of the right-handers associate positive valence with the right side and roughly 60% of the left-handers associate positive valence with the left side). In this context, right-handers are two times more likely than left-handers to associate positive valence with the right and negative valence with the left side. This effect does not substantially change when integrating the country of the participants in our model.

Thus, our results confirm the findings of Casasanto (2009), as we could fully replicate the effects conceptualised in the body-specificity hypothesis. Our relatively large sample and lack of a cultural impact on the respective effects in general suggest that body-specific associations are formed through perceptuomotor experiences, as discussed by Casasanto. More specifically, this means that the dominant and more fluent side is more strongly linked to positive associations than the weaker and less fluent, nondominant side. Accordingly, the nondominant and less fluent side is more strongly linked to negative associations than the dominant and more fluent side. That this effect does not substantially change when integrating the country in our model suggests that space–valance associations are not determined by cultural but rather individual factors.

The study by Casasanto (2009) showed a tendency for the “good is left” mapping in left-handers to be stronger than the “good is right” mapping in right-handers. However, this preference was only significant in one of the five experiments and in the combined data from all participants that showed a left–right bias. Based on this fact and on the substantial difference in the number of right-handers and left-handers, Casasanto points out, that this unexpected finding should be interpreted with caution. However, in his study, he offered two explanations for this potential finding, both of them challenging the body-specificity hypothesis.

Casasanto’s (2009) first explanation states that asymmetries in perceptuomotor experiences might be more salient for left-handers, who often face difficulties due to customs and devices tailored for right-handers. As a consequence, salient perceptuomotor differences could lead to stronger associations between actions performed with their dominant hand and positive valence. Casasanto’s second explanation suggests that the left–right mapping of valence could interact with culture-specific metaphors (e.g., the mental number line; Dehaene et al., 1993) and linguistic expression as “the prime example” (linking primacy and goodness) resulting in a concatenation of left, first, and best. Accordingly, speakers of languages like English could consider the leftmost item as the first and therefore the best. This metaphorical link between left, first, and best could lead to a culturally constructed “good is left” bias among all participants challenging the “good is right” bias in right-handers.

Our data do not show the “good is left” mapping in left-handers to be stronger than the “good is right” mapping in right-handers. In our study, right-handers and left-handers show nearly the same ratio of positive valence to their dominant side. Thus, our findings lend further support to the body-specificity hypothesis in the sense that space–valence associations are determined by perceptuomotor experience rather than culturally formed.

Even though we could confirm the body-specificity hypothesis and could not detect culture-specific influences on space–valence associations in our study, we consider it fruitful to conduct further research in this field. It is plausible to assume that cultural conventions (e.g., the right hand is the “good hand” and the left hand is the “bad hand”) could interact with space–valence associations, even though the latter are not determined by the former (e.g., de la Fuente et al., 2011, de la Fuente, Casasanto, Román et al., 2015a, de la Fuente, Casasanto, Santiago et al., 2015b). Furthermore, our study faced a limitation many studies examining the body-specificity hypothesis encounter. In comparison to right-handers, our sample consisted of a relatively low number of left-handers. Thus, future research could emphasize in finding a more balanced ratio of right-handers and left-handers.

## Data Availability

Data can be found at https://cutt.ly/hBiQr1f

## Footnotes

1 Data, R codes, and supplementary statistical analyses can be found at https://cutt.ly/hBiQr1f

## Code availability

R code can be found at https://cutt.ly/hBiQr1f
